# Examining recruitment differences by race, ethnicity, and age to a decentralized trial of an internet-delivered intervention for sexual health after breast cancer: the WF-2202 SHINE trial

**DOI:** 10.1007/s00520-026-10686-y

**Published:** 2026-04-25

**Authors:** Kelly M. Shaffer, Jillian V. Glazer, Carol A. Kittel, Emily V. Dressler, Eden Wood, Heather Lawson, Jennifer B. Reese, Suzanne C. Danhauer, Shayna L. Showalter, Wendy Cohn, Anita H. Clayton, Kathryn E. Weaver, Glenn J. Lesser, Lee M. Ritterband

**Affiliations:** 1 University of Virginia School of Medicine, PO Box 801075, Charlottesville, VA 22908 USA; 2 Wake Forest University School of Medicine, Winston-Salem, NC USA; 3 Fox Chase Cancer Center, Philadelphia, PA USA

**Keywords:** Age, Breast neoplasms, Cancer survivorship, Clinical trial, Enrollment, Ethnicity, Factorial trial, Internet interventions, Race, Recruitment, Sexual health

## Abstract

**Purpose:**

Achieving demographically representative samples remains a challenge in oncology trials but is essential for generalizable findings. We describe recruitment and enrollment to a decentralized trial for an online sexual health intervention for breast cancer survivors, evaluating differences by race, ethnicity, and age.

**Methods:**

Partnered breast cancer survivors with sexual concerns were recruited to the Sexual Health and Intimacy Enhancement (SHINE) trial (WF-2202, NCT06216574) via the Wake Forest NCI Community Oncology Research Program (NCORP) Research Base. Sites identified potentially eligible survivors; recorded their race, ethnicity, and age; and sent them online eligibility screeners. Eligible, consenting survivors were enrolled.

**Results:**

Screener completion (78.3%, *n* = 557/711), eligibility (66.6%, *n* = 371/557), and enrollment (91.9%, *n* = 341/371) rates were high, but there were discrepancies by race and age. Eligible White survivors were more likely to enroll than eligible survivors from other races (94.4% vs. 87.0%, *p* = .03). Survivors who completed screeners were younger on average than those who did not (age M = 52.6 vs. 54.5, *p* = .05); eligible survivors were also younger on average than ineligible survivors (age M = 51.9 vs. 54.6, *p* = .004). There were no other differences in screener completion, eligibility, or enrollment by race, ethnicity, or age (*p*s > .06).

**Conclusions:**

Differences in screener completion and enrollment by age and race suggest younger, White breast cancer survivors may have had the greatest interest in this trial of an online sexual health intervention. Overall, however, screener completion, eligibility, and enrollment rates were substantially higher for this trial than past clinical trials in oncology, demonstrating the benefits of decentralized trials through the NCORP network.

**Clinical trial registration:**

ClinicalTrials.gov NCT06216574 (Registered: January 22, 2024).

Clinical trial recruitment in oncology is a challenge, with up to 50% of trials closing due to insufficient accrual [1, 2]. At the same time, the accessibility of many clinical trials is limited, with only about 5–8% of cancer patients participating in one [3]. About half of patients are functionally excluded from participating due to travel distance to a trial site [3]; of those remaining, about another half are excluded due to ineligibility [4]. While strong internal validity is important for early phases of treatment evaluation research, constrained eligibility parameters combined with the limited accessibility of many clinical trials risk the development and deployment of interventions with limited effectiveness within real-world clinical care [5].

Patients who are from racial backgrounds other than White, who are Hispanic, or who are older are often underrepresented in clinical trials [3, 6, 7], as these groups are more likely to be excluded based on eligibility criteria and to receive cancer care at underresourced healthcare settings with less infrastructure for clinical trials [8–10]. Indeed, while about 15% of cancer patients are Black/African American, less than 6% of cancer clinical trial participants report this background; similarly, about 13% of cancer patients report Hispanic/Latino ethnicity versus 6% of clinical trial participants [3, 6]. The underrepresentation of older adults in cancer clinical trials is also stark. While about 60% of all new cancer diagnoses are among those 65 or older [11], only about one-third of trial participants fall within this age range [6, 12]. These discrepancies compromise the external validity of cancer care research, prompting multiple leading cancer research organizations to urge greater consideration of clinical trial access [13]. The National Cancer Institute (NCI) developed the NCI Community Oncology Research Program (NCORP) research network to engage community-based oncology practices to increase access to, and representativeness of, oncology clinical trials [14]. Decentralizing clinical trials—moving trial activities to more accessible local locations than traditional tertiary academic healthcare settings, or making all activities remote through digital health strategies—holds significant promise for increasing the representativeness of cancer clinical trial populations [15, 16].

Clinical trials evaluating interventions managing sexual side effects from cancer treatment have multiplied over the past two decades. For breast cancer alone, the number of trials addressing sexual health registered with ClinicalTrials.gov has risen from a total of 33 from the earliest registered trial start date in 1997 through 2010 to a total of 42 starting in 2024 alone. Despite a growing list of evidence-based treatments from these trials [17], and about three-quarters of breast cancer survivors reporting clinically significant sexual symptoms after treatment [18, 19], a minority of survivors ever receive care for these symptoms [20]. These concerns are prevalent across races and ethnicities [21, 22], with cultural differences often impeding clinical communication about sexual side effects [23]. Younger survivors tend to report more sexual problems and more distress about these problems [24, 25], which may help explain why the average age of cancer-related sexual health research samples has tended to be about a decade younger than the average age at diagnosis [17, 26–28], although bias from stereotypes about older adults being uninterested in sex may also contribute [23, 29]. Evaluating the accessibility of clinical trials to improve sexual healthcare for breast cancer survivors is necessary to understand the generalizability of findings and to improve breast cancer survivorship care [13, 30].

Towards this goal, this study reports the recruitment and enrollment of a large, diverse sample of breast cancer survivors to the Sexual Health and Intimacy Enhancement (SHINE) trial, a decentralized clinical trial of Internet-based interventions for cancer-related sexual dysfunction, through NCORP. Differences in recruitment, enrollment, and randomization rates by race, ethnicity, and age are evaluated. Identifying disparities in participation among subgroups of breast cancer survivors will help our team refine study procedures for our follow-up trial and also support other cancer care researchers in designing more equitable clinical trials.

## Methods

### Population and procedures

Full details of the SHINE Trial (WF-2202; NCT06216574) have been previously published [31]; key information is reviewed here. The aim of this factorial trial is to identify a set of fully automated Internet-delivered intervention components expected to improve breast cancer survivors’ sexual distress and dysfunction. Trial procedures and modifications were approved by the NCI Central Institutional Review Board (CIRB). Study materials, including an interactive timeline visualization of recruitment data, are publicly available at https://bit.ly/WF2202-SHINE. De-identified data will be publicly accessible through the NCI National Clinical Trials Network (NCTN)/NCORP Data Archive.

This trial was conducted through the Wake Forest (WF) NCORP Research Base. NCORP is an NCI-funded oncology practice-based research network created to increase access to clinical trials across the US [14]. Only about 10% of NCORP practices are connected with an academic medical center, and 25% are safety-net hospitals [32]. Importantly, all study procedures—including recruitment, screening, consenting, assessment, and intervention—could be completed remotely (e.g., through the Internet), a key recommendation for diversifying trials [15, 16]. A tablet loan program provided cellular data–enabled tablets to any participant in need.

Research staff at NCORP practices identified potentially eligible participants from medical records meeting these criteria: stage 0–III breast cancer diagnosis; ≥ 12 weeks from last cancer treatment (e.g., chemotherapy, surgery, or radiation; ongoing maintenance therapies permitted); no planned cancer treatment in the next 24 weeks; and age ≥ 18. Survivors identified as potentially eligible were invited to complete a confidential online self-reported eligibility screener (available at https://bit.ly/WF2202-SHINE). Individuals were eligible if they self-reported: being female and in an intimate relationship; having at least 1 bothersome sexual symptom [33] that they attributed to their breast cancer or its treatment; being willing to receive study emails; having reliable Internet access (or willing to use a study-loaned data-enabled tablet); no recent serious mental illness; no current couple, marital, or sex therapy; and not being pregnant. The intervention components were only available in English, so all participants needed to be able to read English, as demonstrated by independently completing the English-language online screener. Eligibility criteria were carefully considered to balance our ability to evaluate isolated intervention effects while also affording a generalizable sample from the target population of breast cancer survivors with sexual concerns.

Eligible and interested survivors provided informed consent and were enrolled. Participants were invited by email to complete the online baseline assessment via REDCap, a HIPAA-compliant online survey platform [34]. Automated email reminders and personalized contacts from the recruiting practice encouraged assessment completion; those who did not complete baseline within 21 days of consenting were withdrawn from the study. After completing baseline, participants were randomized to a study condition.

A target of 320 randomized participants was set to provide adequate power to detect intervention effects. Recruitment goals to ensure equitable racial and ethnic representation were set according to (1) incidence rates of breast cancer among women in the US [35] and (2) rates of English proficiency in the US (American Community Survey Data). Given the number of breast cancer survivors treated across NCORP practices and the high prevalence of sexual concerns following breast cancer treatment, we expected to enroll 9–10 survivors per month [31].

### Data sources

#### Race and ethnicity

Potentially eligible survivors’ race and ethnicity were recorded from Cancer Trials Support Unit (CTSU)/Oncology Patient Enrollment Network (OPEN) Portal records, the central registration site for enrolling participants onto NCI-sponsored clinical trials. Participants self-reported their race and ethnicity in the baseline assessment. Where discrepancies between CTSU/OPEN Portal and self-report data existed (n = 20, 6% randomized sample), self-reported data were used.

#### Age

Age was calculated as the difference between the survivors’ date of study registration and date of birth.

### Statistical analysis

Differences in online screener completion, eligibility status, and enrollment by survivors’ characteristics of race (White versus race other than White) and ethnicity (non-Hispanic/Latina versus Hispanic/Latina) were tested with chi-square tests of independence; differences in randomization according to these characteristics were tested with Fisher’s exact tests due to small cell sizes. Differences according to age were tested with independent samples *t*-tests. Tests were all two-sided, *α* = 0.05.

The success of the trial meeting accrual goals by race and ethnicity was evaluated by computing randomization ratios (RR) [30], representing the proportion of individuals in the total randomized sample from a particular group relative to the goal for that group. Ratios > 1 indicate greater representation of a group in the sample than the goal, while ratios < 1 indicate less representation.

## Results

### Recruitment timeline and overview

**Fig. 1:**
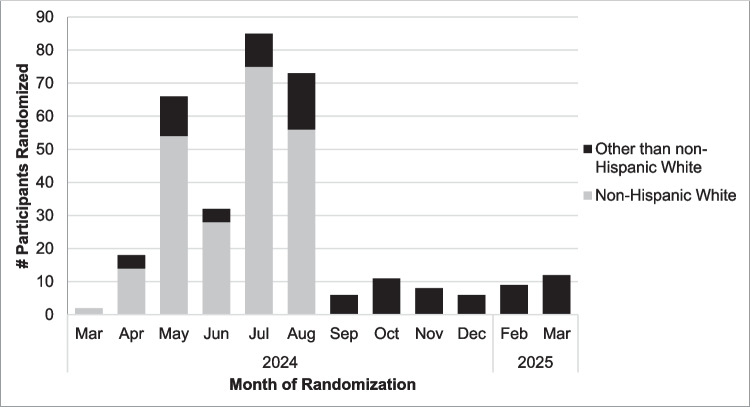
Timeline of participant randomization by race and ethnicity

**Fig. 2:**
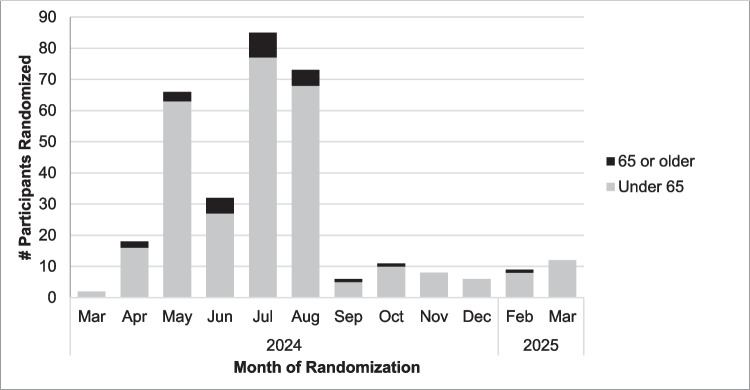
Timeline of participant randomization by age

The WF-2202 SHINE trial opened to accrual on 2024/02/29. By 2024/07/30, 201 survivors had been enrolled, for an average recruitment rate of approximately 40 survivors/month, or about four times faster than anticipated. At that time, however, the enrolled sample was 85.6% non-Hispanic White (*n* = 172), with only 6.0% from a different racial background (*n* = 12) and 6.5% of Hispanic/Latina ethnicity (*n* = 13; unknown race/ethnicity *n* = 4; see Fig. 1 for recruitment timeline by race/ethnicity). A review by members of the study team and the WF NCORP Community Engagement Core identified several potential reasons for demographic disparities, including early study adoption at Midwestern practices with limited patient population racial/ethnic diversity and longer study startup times at practices serving patient populations with greater racial/ethnic diversity. To ensure a representative sample, as of 2024/08/22, enrollment was limited to survivors of a racial background other than White or of Hispanic/Latina ethnicity. As of that date, NCORP practice research staff reviewed survivors’ race and ethnicity as part of preliminary eligibility screening to identify potentially eligible survivors to send online self-reported eligibility screeners. Age was not examined during active recruitment as no goals were set based on this metric (see Fig. 2 for recruitment timeline by age).

In total, 222 NCORP practices opened the trial protocol. Of these, 68 practices (7 designated as minority/underserved) enrolled at least one participant (range 1–43). Participants were enrolled from 26 US states and Washington, DC. The final participant was enrolled on 2025/03/18, totaling about 13 months of recruitment.

### Recruitment trajectory differences by race, ethnicity, and age

The final frequencies and percentages of survivors across the recruitment continuum are described in Table 1. In total, 711 breast cancer survivors were identified through medical record review as potentially eligible to participate. About 70% were White, 90% were non-Hispanic/Latina, and 85% were aged 64 or younger. Of these, 557 (78.3%) completed the eligibility screener. There were no differences in screener completion by race (χ^2^[1]= 0.38, *p* = 0.54) or ethnicity (χ^2^[1]  = 1.19, *p* = 0.27). Age was associated with screener completion: those who completed a screener were about 2 years younger on average (52.64 years) than those who did not (54.48 years; *t*[223] = −2.00, *p* = 0.05).

**Table 1:** Recruitment flow by race, ethnicity, and age

		Race		Ethnicity		Age	
*N* (%)	Total	White	Non-White	Hispanic	Non-Hispanic	≥ 65	≤ 64
Identified potentially eligible	711	506	189	39	651	86	611
Did not complete online screener*	154 (21.66)	105 (20.75)	44 (23.28)	5 (12.82)	140 (21.51)	26 (30.23)	123 (20.13)
Completed online screener	557 (78.34)	401 (79.25)	145 (76.72)	34 (87.18)	511 (78.49)	60 (69.77)	488 (79.87)
Ineligible per online screener^†^	174 (31.24)	120 (29.93)	45 (31.03)	5 (14.71)	160 (31.31)	30 (50.00)	137 (28.07)
Not in an intimate relationship	14 (8.05)	11 (9.17)	3 (6.67)	0 (0.00)	14 (8.75)	5 (16.67)	8 (5.84)
* Excluded due to this criterion alone*	*2 (14.29)*	*2 (18.18)*	*0 (0.00)*	*n/a*	*2 (14.29)*	*0 (0.00)*	*2 (25.00)*
No bothersome sexual symptoms	129 (74.14)	85 (70.83)	36 (80.00)	4 (80.00)	118 (73.75)	24 (80.00)	99 (72.26)
* Excluded due to this criterion alone*	*50 (38.76)*	*33 (38.82)*	*15 (41.67)*	*1 (25.00)*	*46 (38.98)*	*9 (37.50)*	*38 (38.38)*
Sexual symptoms not cancer-related	38 (21.84)	25 (20.83)	13 (28.89)	1 (20.00)	35 (21.88)	9 (30.00)	29 (21.17)
* Excluded due to this criterion alone*	*2 (5.26)*	*2 (8.00)*	*0 (0.00)*	*0 (0.00)*	*1 (2.86)*	*1 (11.11)*	*1 (3.45)*
No regular Internet access & not willing to participate in Tablet Loan Program	2 (1.15)	1 (0.83)	1 (2.22)	0 (0.00)	2 (1.25)	1 (3.33)	1 (0.73)
* Excluded due to this criterion alone*	*1 (50.00)*	*0 (0.00)*	*1 (100.00)*	*n/a*	*1 (50.00)*	*0 (0.00)*	*1 (100.00)*
Recently hospitalized for severe mental illness	3 (1.72)	2 (1.67)	0 (0.00)	0 (0.00)	3 (1.88)	0 (0.00)	3 (2.19)
* Excluded due to this criterion alone*	*0 (0.00)*	*0 (0.00)*	*n/a*	*n/a*	*0 (0.00)*	*n/a*	*0 (0.00)*
Current couple, marital, or sex therapy	86 (49.43)	63 (52.50)	18 (40.00)	2 (40.00)	80 (50.00)	11 (36.67)	72 (52.55)
* Excluded due to this criterion alone*	*32 (37.21)*	*27 (42.86)*	*5 (27.78)*	*0 (0.00)*	*31 (38.75)*	*4 (36.36)*	*27 (37.50)*
Currently pregnant	2 (1.15)	1 (0.83)	0 (0.00)	1 (20.00)	1 (0.63)	0 (0.00)	2 (1.46)
* Excluded due to this criterion alone*	*2 (100.0)*	*1 (100.00)*	*n/a*	*1 (100.00)*	*1 (100.00)*	*n/a*	*2 (100.00)*
Excluded due to other reason^‡^	12	12	0	0	11	1	11
Eligible per online screener	371 (66.61)	269 (67.08)	100 (68.97)	29 (85.29)	340 (66.54)	29 (48.33)	340 (69.67)
Did not enroll*	30 (8.09)	15 (5.58)	13 (13.00)	0 (0.00)	28 (8.24)	1 (3.45)	27 (7.94)
Enrolled^†^	341 (91.91)	254 (94.42)	87 (87.00)	29 (100.00)	312 (91.76)	28 (96.55)	313 (92.06)
Not randomized*	13 (3.81)	9 (3.54)	4 (4.60)	2 (6.90)	11 (3.53)	2 (7.14)	11 (3.51)
Randomized	328 (96.19)	245 (96.46)	83 (95.40)	27 (93.10)	301 (96.47)	26 (92.86)	302 (96.49)

*Note:* Participants excluded from comparisons where data unknown (*N* = 16 for race, 21 for ethnicity, and 14 for age). Percentages are of line above with decreased indent (e.g., randomized % of enrolled [328/341]; enrolled % of eligible [341/371])

^*^Includes both active (expressly declined) and passive (loss to follow-up) refusals and withdrawals

^†^No individuals endorsed exclusion criteria of not being female or not willing to receive study emails

^‡^Individuals not able to be enrolled due to race/ethnicity restrictions or other trial enrollment

Of the 557 survivors completing screeners, 174 (31.2%) were ineligible. About half of these were excluded based on multiple criteria (*n* = 85, 48.9%). The most common reasons for exclusion were as follows: (1) no bothersome sexual symptoms (*n* = 129, 74.1% of ineligible); (2) current couple, marital, and/or sex therapy (*n *= 86, 49.4%); and (3) sexual symptoms not cancer-related (*n* = 38, 21.8%). There were no differences in eligibility by race (χ^2^[1]  = 0.001, *p* = 0.99) or ethnicity (χ^2^[1] = 3.69, *p* = 0.06). Age was associated with eligibility: eligible survivors were about 2.5 years younger on average (51.9 years) than ineligible survivors (54.6 years; *t*[278] = −2.94, *p* = 0.004).

Of the 371 eligible survivors, 341 (91.9%) enrolled. Race was associated with enrollment: while 94.4% of White survivors enrolled, only 87.0% of eligible non-White survivors did (χ^2^[1]  = 4.72, *p* = 0.03). There were no differences in enrollment by ethnicity (χ^2^[1]  = 1.54, *p* = 0.21) or age (*t*[31]  = 0.56, *p* = 0.58).

Of enrolled participants, 328 (96.2%) completed the baseline assessment and were subsequently randomized, while 13 did not complete baseline and were withdrawn (3.8%). There were no differences in randomization status by race (odds ratio [OR] = 1.31 [95% CI 0.29, 4.85], *p* = 0.75), ethnicity (OR = 2.02 [95% CI 0.21, 10.00], *p* = 0.30), or age (*t*[13]  = 0.76, *p* = 0.46).

Of the final randomized sample of 328 participants (age M = 51.9), 30.2% self-identified as from a racial background other than White and/or of Hispanic/Latina ethnicity (see Table 2). The enrolled sample met goals for Black/African American participants (RR = 1.03) and Hispanic/Latina participants (RR = 1.01). The proportion of the randomized sample who reported being from more than one race, or from a different race than those listed, was more than two times greater than anticipated (RR = 2.38). Survivors from American Indian/Alaska Native, Asian, or Native Hawaiian/Other Pacific Islander backgrounds were underrepresented in the sample (RR = 0.31–0.67).

**Table 2:** Randomization rates compared to goals by race and ethnicity

	Not Hispanic/Latina			Hispanic/Latina			Total		
*n* (% total)	Goal	Randomized	RR	Goal	Randomized	RR	Goal	Randomized	RR
American Indian/Alaska Native	3 (< 1%)	1 (< 1%)	0.33	0 (0%)	0 (0%)	n/a	3 (< 1%)	1 (< 1%)	0.33
Asian	13 (4.1%)	4 (1.2%)	0.30	0 (0%)	0 (0%)	n/a	13 (4.1%)	4 (1.2%)	0.30
Native Hawaiian or Other Pacific Islander	3 (< 1%)	1 (< 1%)	0.33	0 (0%)	1 (< 1%)	n/a	3 (< 1%)	2 (< 1%)	0.65
Black or African American	50 (15.6%)	51 (15.5%)	1.00	1 (< 1%)	3 (< 1%)	2.93	51 (15.9%)	54 (16.5%)	1.03
White	217 (67.8%)	229 (69.8%)	1.03	24 (7.5%)	16 (4.9%)	0.65	241 (75.3%)	245 (74.7%)	0.99
More than One Race / Other	8 (2.5%)	15 (4.6%)	1.83	1 (< 1%)	7 (2.1%)	6.83	9 (2.8%)	22 (6.7%)	2.38
Total	294 (91.9%)	301 (91.8%)	1.00	26 (8.1%)	27 (8.2%)	1.01	320 (100%)	328 (100%)	1.00

*RR*, randomization ratio = (Randomized *n*/328)/(Goal *n*/320)

## Discussion

This decentralized trial of an online sexual health intervention for breast cancer survivors conducted through the WF NCORP Research Base recorded swift recruitment that exceeded expectations and resulted in a sample largely representative of English-speaking breast cancer survivors in the US. There were no differences in screener completion or eligibility by race or ethnicity; however, older survivors were less likely to complete the online eligibility screener and to be eligible to participate relative to younger survivors. As for enrollment, while there were no differences according to age or ethnicity, eligible White survivors were more likely to join the study than those from another race, although enrollment rates were high across groups (≥ 87%). These differences in trial access and enrollment notwithstanding, findings suggest that this Internet-based sexual health intervention trial was accessible, feasible, and desirable to breast cancer survivors within a prominent oncology research network.

The WF-2202 SHINE trial was deliberately designed to be widely accessible to survivors and feasibly implemented at NCORP practices. Considerations included making it possible to complete all research activities remotely (e.g., remote consenting approved, no in-person study visits required) and carefully selecting eligibility criteria to balance both internal and external validity concerns [15]. These design choices likely contributed to the trial’s successful accrual, meeting or exceeding benchmarks from past trials of Internet-based sexual health programs for cancer survivors (i.e., eligible of screened: 67% SHINE vs. 40–80% past trials; enrolled of eligible: 92% vs. 55–77%) [36–38]. It is also worth noting that only one survivor was excluded as a result of not having Internet access and being unwilling to participate in the study tablet loan program, which was only used by two participants. These successes underscore the clear interest in, and accessibility of, decentralized trials of digital health interventions for sexual health side effects from cancer [16].

This trial recruited nearly four times faster than anticipated through the first several months, although the recruitment of non-Hispanic White participants quickly outpaced those from other racial and ethnic backgrounds. This pattern of enrollment has also been documented among other rapidly accruing US national clinical trials [39]. Sites that serve underrepresented patients often encounter more barriers to study startup, including stricter regulatory demands and fewer resources dedicated to research infrastructure [39–41]. Notably, when SHINE trial recruitment was limited to survivors from racial backgrounds other than White and to Hispanic/Latina survivors, we continued to meet our original trial enrollment goal of accruing about nine survivors per month. In keeping with prior recommendations [30], establishing and tracking enrollment goals were critical to our ability to secure a representative sample and account for slower, but steady, accrual of underrepresented survivors.

There were no differences by race or ethnicity in terms of the accessibility of the SHINE trial, as shown by comparable rates of screener completion and eligibility; however, eligible White survivors were about two times more likely to enroll in the trial relative to eligible survivors from other races. These findings differ from those of prior studies showing no differences in cancer clinical trial enrollment rates by race [42, 43]. It is worth noting, however, that even this lower enrollment rate among survivors of racial backgrounds other than White (87%) exceeded overall enrollment rates from past Internet-based sexual health survivorship studies (55–77%) [36–38]. Enrollment goals were ultimately met for Black/African American survivors and Hispanic/Latina survivors, but Asian American, Native Hawaiian, and Pacific Islander (AANHPI) survivors were significantly underrepresented in the sample. One contributing factor may be the largely community-based practice setting of recruitment through NCORP, with one meta-analysis showing that Asian cancer survivors were half as likely to agree to a clinical trial when offered in a community versus academic hospital setting [42]. While there has been limited research on sexual well-being among female AANHPI cancer survivors, available studies suggest this group may also be less likely to complete patient-reported outcomes about sexual functioning than White patients [44, 45], whether as a result of cultural differences in norms and attitudes, or due to experiences of discrimination in the healthcare system [45, 46].

While the average age of the enrolled sample was comparable with those from prior sexual health interventional trials among breast cancer survivors [17], there were discrepancies in trial accessibility by age from the point of pre-screening. Only 18% of the survivors sent eligibility screeners were aged ≥ 63, the median age of breast cancer diagnosis in the US [26], which may reflect assumptions that older women may be uninterested in a sexual health intervention [29]. This discrepancy was compounded by older adults being less likely to complete the online screener, and those who did were almost twice as likely to be ruled ineligible relative to younger survivors. Older survivors were commonly excluded due to not endorsing bothersome sexual symptoms, or that their symptoms were unrelated to their breast cancer, fitting with prior literature [24, 25]. Importantly, among screened and eligible survivors, there was no difference in the rate of enrollment by age. This finding suggests that the trial was equally of interest to eligible survivors regardless of age, which contrasts with findings that older survivors showed lower trial enrollment across the NCI Community Cancer Centers Program [43]. While older adults may be less likely to seek out and adopt digital health interventions, those who do show strong engagement that rivals or surpasses that of younger users [47, 48].

Results from this analysis should be considered in light of limitations. First, only those survivors who were identified as potentially eligible per medical record review could be evaluated. Data were not recorded for survivors who were not screened, were found ineligible based on preliminary screening, or may have preemptively declined screening, so discrepancies at these levels cannot be determined. Furthermore, this analysis was restricted to factors available from CTSU/OPEN (i.e., age, race, ethnicity), yet other factors like rurality or socioeconomic status may play more significant roles in clinical trial access [49, 50]. Evaluating differences by race between White survivors and all those from other racial backgrounds may obscure discrepancies between races, highlighting the need for further large-scale, diverse trials for more nuanced analyses. This analysis is of a single clinical trial conducted through the unique setting of the NCORP research network with a fully remote design, and may not be representative of recruitment patterns for trials in other cancer disease sites, other trial designs, or interventions targeting other symptoms. Lastly, this trial was limited to English-speaking participants; cultural and linguistic adaptation of the intervention for Spanish-speaking survivors is a future research objective.

Findings from the recruitment and enrollment of breast cancer survivors to the WF-2202 SHINE trial demonstrate strong demand for clinical trials for Internet-based sexual health interventions, which can rapidly achieve a demographically representative sample through nationwide oncology research networks. Factors that may have supported successful accrual include the trial’s decentralized design, limited eligibility criteria balancing internal and external validity, and that sample demographic goals were set and tracked. Early discrepancies in trial access and enrollment among survivors by race and ethnicity underscore the importance of proactive recruitment oversight to support equity across the recruitment pipeline. Further evaluation is warranted to determine why older survivors were underrepresented among those approached for screening, as well as why White survivors were more likely to enroll than survivors from other racial backgrounds, to ensure this intervention is equally appealing and effective across all breast cancer survivors. Overall, however, the markedly high rates of screening completion and enrollment across survivors highlight the unmet need for sexual healthcare in breast cancer survivorship and the enthusiasm for research in this domain.

## Data Availability

Study materials are publicly available at https://bit.ly/WF2202-SHINE. De-identified data will be publicly accessible through the NCI NCTN/NCORP Data Archive.
